# Addressing Training Gaps: A Competency-Based, Telehealth Training Initiative for Community Health Workers

**DOI:** 10.1089/tmr.2023.0007

**Published:** 2023-06-16

**Authors:** Laura Porterfield, Victoria Warren, Vanessa Schick, Shannon Gulliot-Wright, Jeff R. Temple, Elizabeth M. Vaughan

**Affiliations:** ^1^ Department of Family Medicine, University of Texas Medical Branch (UTMB), Galveston, Texas, USA.; ^2^ Sealy Institute for Vaccine Sciences, UTMB, Galveston, Texas, USA.; ^3^ Department of Health and Human Performance, University of Houston, Houston, Texas, USA.; ^4^ Management, Policy, and Community Health, School of Public Health, University of Texas, Houston, Texas, USA.; ^5^ School of Nursing, UTMB, Galveston, Texas, USA.; ^6^ Department of Internal Medicine, UTMB, Galveston, Texas, USA.; ^7^ Department of Medicine, Baylor College of Medicine, Houston, Texas, USA.

**Keywords:** telehealth, Community Health Workers or CHWs, training, competency, education, public health

## Abstract

**Background:**

To overcome vast variations in Community Health Worker (CHW) training, investigators for the CHW Core Consensus Project (CCCP) derived three types of CHW (Category 1, 2, 3) and established competencies for each category. However, studies are needed that implement these competencies in real-world settings.

**Objective:**

Using the six competency domains of the CCCP as a theoretical backbone, we developed and evaluated a training for *Category 1* CHWs, individuals whose focus is on community outreach and advocacy.

**Methods:**

We developed five telehealth-based, bilingual (Spanish/English) training sessions and implemented them among *Category 1* Latino(a) CHWs. We measured the number of CHWs who achieved ≥70% correct on a domain-based posttest, attendance, and qualitative feedback.

**Results:**

All (18/18) CHWs achieved at least 70% on the posttest (mean: 93.7%; range 73.3–100%). Training attendance was 98.9%. Using a six-point scale, CHWs reported high levels of satisfaction overall (5.72 ± 0.57/6.0), with telehealth (5.72 ± 0.58/6.0), effectiveness of teaching strategies/methods (5.89 ± 0.32/6.0), achieving training objectives (5.96 ± 0.15/6.0), knowledge improvement (5.72 ± 0.57/6.0), and interest (5.78 ± 0.43/6.0).

**Conclusion:**

We successfully developed and evaluated a bilingual training program for *Category 1* CHWs to address core competency gaps. High CHW attendance reinforces the value of telehealth modalities and their potential to increase the *reach* for CHW training. To overcome gaps in chronic disease training, investigations are needed to address additional CHW trainings.

**Trial Registration:**

NCT04835493.

## Introduction

Community Health Workers (CHWs) are individuals with close cultural and/or linguistic ties with the people that they serve.^1^ They have a unique capacity to connect individuals with health services, support, and culturally appropriate health information, both as health care members and as residents of the communities.^2,3^ The CHWs are playing a growing role in public health research.^4^

They show particular promise to promote health equity in minority communities that experience linguistic barriers, disadvantages due to structural racism, economic hardships, and disparities in chronic disease burden.^5^ The CHWs have demonstrated efficacy as a cost-effective approach to improve many conditions and disease outcomes in populations experiencing disparities.^2^ The CHW-mediated initiatives have been effective in numerous interventions, including behavior change, use of preventive and screening services, chronic disease monitoring, mental health, and quality of life.^2,3,6^

Despite CHWs' key role in advancing the health of vulnerable communities, there is a major gap in structured, robust CHW training studies.^7^ A few US states regulate CHW training and support; only five states require certification or training, seven have advisory bodies, eight define a scope of practice, and six authorize a standard curriculum with core skills.^8^

Even in states such as Texas with a rigorous 160-h CHW certification process, critical health care concepts including telehealth, Health Insurance Portability and Accountability Act (HIPAA), and Protected Health Information (PHI) are not included in the curriculum.^9^ Training and evaluation of its effectiveness are complicated by diversity in CHW roles, supervision, interventions, work environment, resources, and health system support.^3–7,10–13^

A systematic review of 61 studies of CHW interventions found striking variations in the approach, duration, and content of training, with over half of the studies providing incomplete descriptions of training, and 13% providing no information at all.^2^ Further, CHWs are often racial and ethnic minorities whose first language is not English, but there is a paucity of training available other languages.^14,15^

To provide structure for training, Covert et al. established standardized core competencies directly associated with CHWs' workforce.^16^ Consistent with the community health workforce framework methodology, investigators for the CHW Core Consensus Project (CCCP) gathered a national expert panel of 15 members to conceive a list of measurable, validated competencies framed into six major domains.^16^ Investigators also delineated the CHW workforce into three categories based on their training, work setting, scope of practice.

The more specialized a CHW, the higher the category. *Category 1* CHWs work in community-based organizations, are trained in health outreach and advocacy, and scope of practice includes providing health information and promoting awareness. For example, they may lead a community vaccine campaign or go on home visits to navigate food accessibility. *Category 2* CHWs may also work in community organizations but receive specialized training regarding chronic conditions to initiate targeted community health activities and engage stakeholders.

*Category 3* CHWs often work in more structured outpatient settings, such as clinics, and have disease-specific training (i.e., diabetes or cancer) to coordinate therapies, navigate care, and provide education. The CHWs may shift between categories; the most specialized group, *Category 3* CHWs, would likely need training in all category(ies) to successfully implement work.^16^

Competency-based education offers a measurable, practice-based approach to the education and assessment of health professionals, including CHWs.^16,17^ Competencies are made up of entrustable professional activities and tasks that a given professional will be expected to carry out on completion of training.^18,19^ However, fewer than 15% of CHW initiatives apply a competency-based training approach.^2,17^ We utilized the CCCP for the theoretical framework to develop and implement a bilingual (English/Spanish) training program for *Category 1* CHWs. We evaluated the intervention's impact with quantitative and qualitative survey data, including levels of CHW knowledge, attendance, and satisfaction.

## Methods

### Training development

#### Setting and participants

We evaluated a *Category 1* CHW training program (May–November 2022) occurring in the greater Houston area for CHWs serving a low-income, Latino(a) populations at two community clinics in different health systems. At each site, we aimed at recruiting a pre-specified number of CHWs based on needs for upcoming clinic initiatives (site 1: *n* = 12, site 2: *n* = 6). We recruited from a pool of clinic volunteers, nearby churches, and community sites.

The CHWs were self-identified as Latino(a)s and were fluent in Spanish (or bilingual in Spanish and English) and certified by the state of Texas. For CHW state certification, the Texas Department of State Health Services requires 160 h of coursework or 1000 h of community service in the prior 3 years, followed by 20 h of continuing education biennially to maintain certification.^9^ This study was approved by the Institutional Review Board at Baylor College of Medicine.

#### Theoretical framework

To establish training for *Category 1* CHWs, we utilized the community health workforce framework from the CCCP as the theoretical backbone.^16^ The six competency domains from the project provided the framework for our curriculum: (1) Assessment in Session 1, (2) Communication in Session 2, (3) Diversity and Inclusion also in Session 2, (4) Disease Prevention and Management in Session 3, (5) Professional Practice in Session 4, and (6) Community Health Practice in Session 5.^16^

To obtain curriculum content, investigators reviewed the literature to obtain five evidence-based, sessions.^5,8,9,11,16,20^ Table 1 illustrates the theoretical model for training as they link to the curriculum details: objectives, competency domains, formant, content, continuing education hours, and additional assignments. The Texas Department of State Health Services approved this training for 5 h of formal CHW continuing education credit.

**Table 1.** Theoretical Framework with Detailed Curriculum for *Category 1* Community Health Worker Training^16^

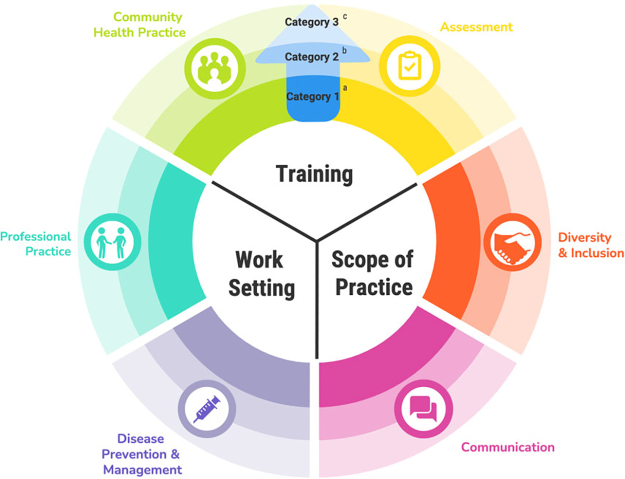							
***Session***	***Title***	***Learning objectives***	***Competency domain***	***Format***	***Content***	***CEUs** ^d^*	***Additional assignments** ^e^*
1	How CHWs Advocate	Discuss your interests Define CHW List roles of CHWs	Assessment	In-person	Community partners CHW role examples State CHW certification requirements	1	Ensure internet connection and device connectivity
2	The Importance of CHW Advocacy	Review CHW value and roles Describe telehealth logistics	Diversity and Inclusion Communication	Tele	CHW definitions Cultural sensitivity and disparities Making referrals Supporting CHWs Practicing with telehealth	1	Chapter 1, 2
3	Outreach:Working in Health care	Recognize care gaps Define HIPAA and PHI Identify ways to recruit patients	Disease Prevention and Management	Tele	System vs. provider vs. patient-related gaps Applying HIPAA and PHI to daily work Participation is voluntary	1	Chapter 3, 4
4	Outreach-Site Set-up	Increase awareness about eligibility Discuss clinic policies and procedures	Professional Practice	Tele	Clinic eligibility Medication eligibility Specific site logistics for set-up	1	Chapters 5, 6
5	Outreach Real-world Issues	Identify needs in Maslow's pyramid Decipher emergent vs. non-emergent situations Recognize learning styles	Community Health Practice	Tele	Details of Maslow's pyramid Triaging emergencies Building trust Stages of changes Auditory, visual, tactile learners	1	Chapters 7, 8 Evaluation Post-test

^a^ Category 1 CHWs have training in health outreach and advocacy, work in community organizations, and focus on outreach and awareness.

^b^ Category 2 CHWs have training in chronic conditions, work in primary care clinics/community organizations, and focus on access to care and community relationships.

^c^ Category 3 CHWs have disease-specific training, work in community clinics, and focus on access and disease-specific interventions.

^d^ Texas Department of State Health Services Continuing Education Units.

^e^ Reading assignments from Tomando Control de Salud.^20^

CHW, Community Health Worker; HIPAA, Health Insurance Portability and Accountability Act; PHI, Protected Health Information.

### Training implementation

Texas-certified CHW-Instructors taught an interactive lecture-style training in Spanish, which was overseen by a bilingual physician. At the end of sessions, instructors assigned additional reading assignments from *Tomando Control de Su Salud*, a Spanish text that assists non-medical readers in self-management through goal setting, action planning, decision making, and problem-solving.^20^ Through OhMD, a HIPAA-compliant, encrypted group text-messaging system,^21^ CHWs shared key points learned from their readings during the week. Instructors responded to texts to encourage learners in their work. English and Spanish versions of the PowerPoints may be found online at mipromotordesalud.org. All trainers were part of a study team.

There were five required CHW training sessions. The first session occurred in-person for 1 h, and the next four were weekly via telehealth for 4 weeks 1 h each evening 4 months later. This time gap provided the opportunity to identify and address potential issues with telehealth training, including internet access, ability to log onto ZOOM, and device needs. The expected time commitment for CHWs was 4 h per week. Based on prior research, we defined telehealth as utilization of a technology-based virtual modality to provide health information.^22^

#### Technology requirements

Before the COVID-19 pandemic, we found that offering trainings virtually substantially expanded the ability to reach CHWs without sacrificing knowledge outcomes.^23^ Instructors used telehealth (ZOOM) as the virtual training platform for the current study. The training required internet access with broadband 3G or 4G/LTE and minimum bandwidth of 600 kbps.^24^ We provided a tablet with a camera, microphone, and ability to view word-processing documents to CHWs who did not already have a device.

In our prior studies, CHWs had pre-existing internet access in their homes; however, in the case that they lost access, we directed to sites in the community where they could gain access (i.e., churches, community centers, libraries). To enhance security, we sent direct password protected links, maintained updated software versions, and followed *Best Practices*.^24^

### Training evaluation

Our primary outcome evaluated end-of-course CHW knowledge. Secondary outcomes included attendance and qualitative measures of CHW satisfaction.

#### CHW knowledge

We derived one posttest covering the training content that consisted of six case studies with 2–3 questions each, totaling 14 questions. Case study evaluation used a mix of quantitative, multiple-choice and qualitative, short answer responses. To derive the posttest, we used evidence-based resources to address each of the six competency domains: Case 1 (Communication), Case 2 (Assessment), Case 3 (Professional Practice), Case 4 (Community Health Practice), Case 5 (Disease Prevention and Management), and Case 6 (Diversity and Inclusion).^16,25^

Answers were evaluated for by total overall score and for each individual. We defined training competency as an individual achieving at least 70% correct answers.^26^ A copy of the posttest may be found in Supplementary Appendix Table SA1. Pretests were not administered to avoid affecting posttest performance through familiarity with questions.^27^

#### Attendance

We defined attendance as a visually confirmed presence with camera on for at least 45 min of the 1-h training session.

#### Evaluations

The CHW satisfaction was measured through the 15-item Texas Department of State Health Services survey that includes 12 questions assessing training content (6-point Likert scale anchored by 1 = poor and 6 = excellent) modified for this study (Supplementary Appendix Table SA2).^9^ The remaining three questions were qualitative and dealt with relevance, future topics, and additional comments. The CHWs completed the evaluation after completing the training series.

## Results

As shown in Table 2, CHWs (*n* = 18) were 47.6 (±11.6) years old, mostly female (88.9%), and bilingual (77.8%). At baseline, the majority were employed in administrative/office (38.9%) or educational/ministry (33.3%) work. Most worked full-time (61.1%), almost half had completed college (44.4%), and nearly all were born outside the United States (Central America 72.2% and South America 22.2%).

**Table 2.** Demographics of Community Health Workers (CHWs) Participating in Category 1 CHW Training (*n* = 18)

*Variable*	****n*** (%) or mean (SD)*
Age (years)	47.6 (±11.6)
Gender	
Female	16 (88.9%)
Male	2 (11.1%)
Employment history	
Administration/office	7 (38.9%)
Education/pastoral	6 (33.3%)
Domestic	2 (11.1%)
Other	3 (16.7%)
Work commitments	
Full-time	11 (61.1%)
Part-time	7 (38.9%)
Education	
High school	3 (16.7%)
Some college/post- secondary program	7 (38.9%)
Completed college	8 (44.4%)
Language	
Bilingual (English/Spanish)	14 (77.8%)
Spanish only	4 (22.2%)
Country of birth	
United States	1 (5.6%)
Central America	13 (72.2%)
South America	4 (22.2%)

SD, standard deviation.

### Posttest outcomes

**FIG. 1.**
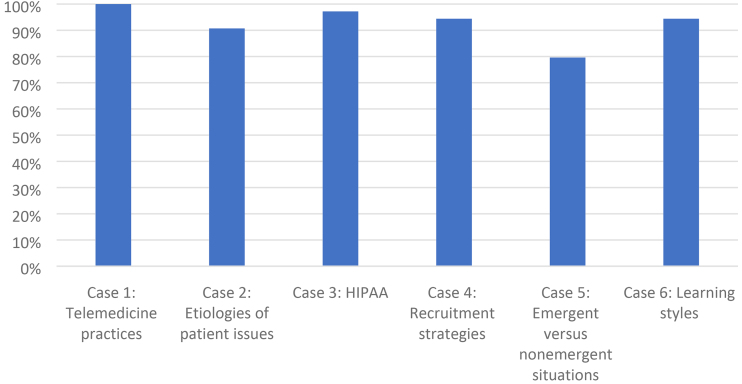
CHW posttest outcomes by case study area (*n* = 18 CHWs). Correct responses on the six case studies were as follows: case study 1 (telehealth practices, 100%), case study 2 (etiologies of patient issues, 90.7%), case study 3 (HIPAA, 97.2%), case study 4 (recruitment strategies, 94.4%), case study 5 (emergent vs. non-emergent situations, 79.6%), and case study 6 (learning styles, 94.4%). CHW, Community Health Worker.

As shown in Figure 1 , all CHWs post-test was scored above a 70%, with the total scores ranging from 73% to 100% (mean = 93.7%) and the overall correct responses on the six case studies. Case study 1 (telehealth practices) resulted in the most correct responses (100%); case study 5 (emergent vs. non-emergent situations) had the fewest correct responses (79.6%).

### Attendance outcomes

Seventeen of the 18 CHWs were present for each of the five sessions. One CHW missed one of five sessions, resulting in a mean attendance of 98.9%.

### Qualitative outcomes: CHW satisfaction

Survey results (Table 3) suggest that CHWs were highly satisfied with the training program overall. Specifically, they indicated satisfaction with telehealth as a venue for training, instructor expertise, meeting objectives including HIPAA, PHI, and determining emergent versus non-emergent situations, relevance, and increased knowledge and interest.

**Table 3.** Texas Department of State Health Services Community Health Worker (*n* = 18) Satisfaction Survey Results (Rating Scale 1–6, Where 1 Is Poor, and 6 Is Excellent)^9^

*Evaluation component*	*Mean ± SD*
1. Circle the number that best represents your overall rating of the program	5.72 ± 0.57
2. Rate the expertise of the instructor(s)	5.89 ± 0.32
3. Were the teaching strategies/methods effective?	5.89 ± 0.32
Did the presentation meet the following objectives:	
4. Discuss the value of CHWs	5.83 ± 0.38
5. Recognize gaps in access to care in your community	5.50 ± 0.86
6. Define HIPAA and PHI	5.78 ± 0.42
7. Determine emergent vs. non-emergent situations	5.89 ± 0.32
8. Were the objectives relevant to the overall purpose of the presentation?	5.83 ± 0.38
9. Was telehealth appropriate for learning?	5.72 ± 0.58
10. Did the presentation increase your level of knowledge in the subject?	5.72 ± 0.57
11. Did the presentation increase your level of interest in the subject?	5.78 ± 0.43
12. Was the subject matter of the course relevant to your work?	5.78 ± 0.43
13. Describe how you will you use the information presented in your practice *- I will use it in our next diabetes management program to be able to identify emergent situations* *- I will use this information on my patients, and on my own family, especially those with chronic disease, to help them to have a better healthy diet. The are many topics we have analyzed and learned, like how a patient can know when to contact his/her doctor in a case of a possible emergency.* *- In many ways—to mention one, asking questions to determine if a situation is emergent or not* *- I will be better informed so I can help patients better.* *- My current work requires customer service information and learning is very useful in my daily work activity as I interact with customers every day. I feel more knowledgeable about topics I was not aware of such as confidentiality on personal information. I also can use this information on people I come across on a daily basis.* *- This information will help me to identify the best way to assist a patient, recognize if there is a failure in the system, or a barrier between the doctor and the patient, and be able to communicate with the patient respecting his privacy and confidentiality.* *- I will use it to train CHWs and teach patients.* *- This information will help make better decision when communicating with the (patients)* *- I will use at work and reenforce HIPAA.* *- I will use this information as a social support for my patients to recognize care gaps and how to advocate.*	
14. List topics for future education programs. *- Ideas for medication compliance* *- 1) Create strategies about prevention and chronic disease control. 2) Learn about risk factors in other parts of the community, like children and adolescents. 3) Try to investigate how to inform and activate, such as having a well-prepared and proactive health team in the field of health.* *- Medications, mental health, and depression* *- Depression, Anxiety, Nutrition, Self-improvement, communication* *- Diet and exercise, self-care* *- Depression, Mental Health, Cancer* *- Mental health* *- I do not have any right now.* *- Nutrition, high blood pressure* *- Mental health* *- Physical, mental, emotional damage from diabetes* *- I would like to know more about high blood pressure* *- Basic computer classes* *- Cardiovascular disease and stroke* *- Get to know different computer programs* *- Student-led learning*	
15. Additional comments *- Excellent class and clear points* *- Talking about health topics, there is so much to continue studying and analyzing. All of these lessons are absolutely, wonderful and it will be a great help from God to keep going and reach the most vulnerable such as children and teens.* *- Everything good. I'm very excited to be part of the project.* *- This has been a great tool for me. I am learning so much information that I can apply to my daily activities.* *- Excellent presentation and very useful* *- Enjoyed class* *- Good and patient presentation* *- Thank you for your teaching and your time. Each of the classes have been very enriching.* *- The topics they teach us are very pertinent, to better enhance the work* *- I am very excited about these classes. I think we have a lot to learn to help people who need it. Thank God for each of you. They are an inspiration for us who are starting. I personally admire them. Thank you* *- These topics are interesting and keep learning.* *- Thank you for the learning you provide us.*	

In response to the open-ended questions, CHW comments were positive, and their responses were thematically similar. When asked how CHWs would use the information in their practice responses included, “help make better decision(s) when communicating with the clients,” to “reenforce HIPAA,” and “to determine if a situation is emergent or not.” When asked to list topics for future education programs, they commonly listed mental health, nutrition, and medications. The CHWs were also given the chance to provide any additional comments on the program. They provided overall positive feedback, such as: “excellent class and clear points” and “this program has been a great tool for me. I am learning so much information that I can apply to my daily activities.”

## Discussion

We addressed gaps in CHW training by successfully designing, implementing, and evaluating the acquired knowledge, feasibility, and satisfaction of a bilingual, competency-based telehealth training program for *Category 1* CHWs. We found that CHWs had high levels of knowledge on the post-test, high rates of attendance, and reported satisfaction.

Health care organizations are increasingly expected to assess and address health disparities and patient health-related social needs.^28^ The CHWs expand the ability of health care teams in this area and are likely to play an increasingly important role in health care.^2,5,29^ Given the likely increase in CHWs, it is vital for both patient safety and effective research that we adequately train and account for CHW competencies. Competency-based education offers a measurable, practice-based approach to the education and assessment of health professionals, including CHWs.

However, there is a gap in its implementation for CHWs based on their scope of work.^2,17^ Our bilingual curriculum addresses competency gaps by targeting to *Category 1* CHWs and using an established framework, which may be used as foundational training for more specialized *Category 2* and *Category 3* CHWs.^16^

In addition, the study incorporated specific training for key topics. For example, COVID-19 provided a worldwide awareness of telehealth's ability to increase the *reach* of training platforms in low-income areas; however, with increased use, gaps in training for CHWs are now apparent, and it is essential for training programs to include digital literacy for trainees to use and navigate an increasing number of platforms.^30,31^

In this study, virtual meetings were likely a major contributor to attendance, yet equipment and training was critical in making this successful. In addition, the curriculum included fundamental topics needed to work in the medical field that are not standard in CHW education (i.e., PHI, recruitment skills, HIPAA, social determinants of health, Maslow's hierarchy of needs, and teaching strategies). A few US states have rigorous CHW certification requirements, yet even those that do often omit these topics.^9,14^

Further, the case-study format for a posttest enabled investigators to see strengths and weaknesses of CHW knowledge. For instance, we recognized that emergent versus non-emergent situations scored lower than other topics, which may be a reflection that CHWs often do not have a medical background and flagged this as a need for ongoing education in future trainings.

Study limitations include a predominance of Latino(a) participants and in a US setting, which may restrict generalizability of results to other populations or to global contexts. Significant geographic, racial, and ethnic disparities in broadband access exist in the United States, and some populations that could particularly benefit from the CHW training described in our study may be limited by the lack of broadband access.

In addition, the majority of CHWs in the study reported having completed some or all college, which may represent a higher average education level than what is typically perceived for CHWs, which could confound results on CHW knowledge testing. However, due to lack of standardized reporting on CHW educational levels, it is difficult to ascertain whether the educational backgrounds of the participants in our study are atypical for CHWs.^4,6^

Future investigations are warranted that are larger, in other ethnicities, and locations. The 18 individuals in our study were part of initiatives in the clinic that had a set number of CHWs assigned to patients. Although we found a high level of CHW interest, these set patient numbers hindered us from recruiting more CHWs. In addition, investigations are needed to develop competency-based training for *Category 2* and *Category 3* CHWs.

Further, data are needed to address CHW training sustainability. Although there have been shifts in the past decade in improving CHW certifications, in order for these key players to been seen as part of the disciplinary team, as are physicians, nurses, therapist, social workers, and numerous other important members, national standardization of training is needed and including them as a line item in the budget is critical.^32^ Until then, CHWs are more likely to be involved in patient care through grant funding with set end dates rather than active team members.

### Public health implications

We designed and implemented a competency-based training for *Category 1* CHWs framed by the Core Consensus Project and found high rates of attendance, knowledge acquisition, and satisfaction. As CHWs continue to play an emerging and vital role in patient care, the pursuit of health equity and attention to competency-based training will be critical. Future initiatives are needed to implement CHW training in chronic disease to continue to overcome training gaps.

## Supplementary Material

Supplemental data

Supplemental data

## Supplementary Material

Supplementary Appendix Table SA1

Supplementary Appendix Table SA2

## Abbreviations Used

CHW: Community Health Worker
CCCP: CHW Core Consensus Project
HIPAA: Health Insurance Portability and Accountability Act
PHI: Protected Health Information
SD: standard deviation
