# Supplementary material for: Addressing Training Gaps: A Competency-Based, Telehealth Training Initiative for Community Health Workers
Source: Telemed Rep. 2023 Jun 16;4(1):126–34. doi: 10.1089/tmr.2023.0007 (PMC10282968; doi:10.1089/tmr.2023.0007)
Supplement: Supplemental data [file Suppl_AppendixTableSA1.docm]

| **APPENDIX 1.** *Category 1* Community Health Worker (CHW) posttest consisting of six case studies with a total of 14 questions | | |
| --- | --- | --- |
| **Case 1** | | |
| You are attending the weekly ZOOM training. Which picture best describes how you hope your other CHWs look while listening to the presentation?^25^ | | 1. Circle your answer:  A B C D |
| A  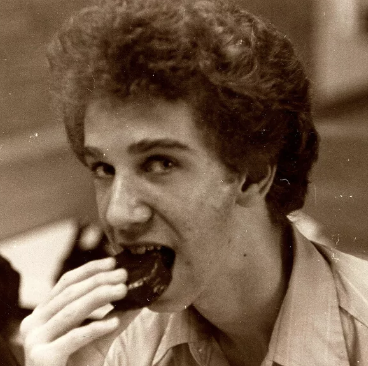 | B  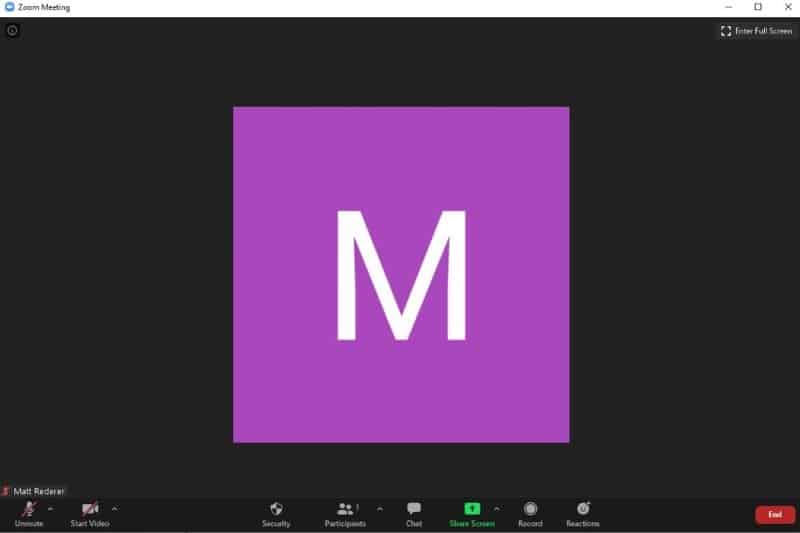 | Write 2 reasons why you chose your answer.  2.  3. |
| C  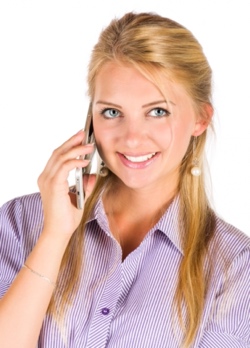 | D  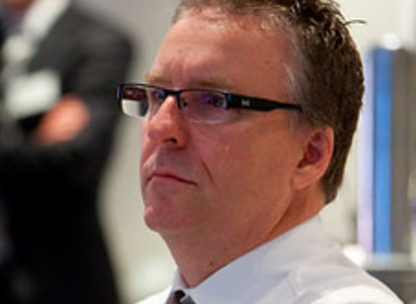 |  |
| **Case 2** | | |
| Your patient cannot get his refills. The clinic tells you that that they called your patient, but he did not answer and his voicemail is not working.  The physician did not understand that he needed refills at the last visit.  The clinic was closed for a few days, and they are now behind in their schedule but they made an appointment for next Monday at 11AM.  Write one patient, one system, and one provider issue that were barriers to your patient getting his medication? | | 4. Patient issue: |
|  |  | 5. System issue: |
|  |  | 6. Provider issue: |
| **Case 3** | | |
| You are a new CHW and call your patient, Mario. He is not home so you leave a message on Mario’s cell phone, “your doctor told me that your blood sugars are getting better and we look forward to seeing you at your appointment next Tuesday at 10AM.”  His friend (Jose) calls you back from Mario’s phone and asks if everything is OK. You ask if Mario has been taking his blood pressure and diabetes pills. Jose thinks he has but is not sure, so he texts another friend, and finds out he has been taking his medications.  You tell Jose not to let Mario forget his appointment for diabetes on Tuesday. You think the doctor might need to do a prostate exam at the appointment so to come a little early. | | Write 2 HIPAA violations from this case  7.  8. |
| **Case 4** | |  |
| You are assigned 100 patients to call this week for the outreach program. You are convinced that you alone will get all 50 patients for the program. You cannot find the script anywhere so you decide to “wing it”.  The first 15 numbers are disconnected. The next 10 patients tell you “no”, but you tell them they better show up “or else”. The next 15 patients tell you ”yes”.  You take a break for coffee. You feel that you deserve it….or need it. You reflect on your experience calling patients: | | 9. Name 1 thing you did right:  10. Name 1 thing you could do differently: |
| **Case 5** | |  |
| Alma’s patient, Miguel, has type 2 diabetes, obesity, and high blood pressure. Miguel calls Alma and tells her that part of his face is numb. He wonders what he should do.  When his wife hears Miguel talking to Alma, she takes to phone and tells her that this just happened 10 minutes ago, it has never happened before, he refused to call the clinic or go to the Emergency Department/Room until he talked to Alma. He hasn’t been taking his blood pressure pills and their home readings have been high. | | 11. Circle your answer. Is this likely:  Emergent or Non-Emergent |
|  |  | 12. What is 1 thing Alma should do? |
| **Case 6** | |  |
| You sit next to a patient during diabetes program. He is quiet but answers your questions when asked. He can tell you word for word what you said!  During class he is very engaged listening to lectures. At the end you ask if there are any questions and he responded, “No, I hear you. I understand.”  You are trying to find a way to determine if you are effectively teaching him and meeting his need and think figuring out his learning style might be a good start. | | 13. What do you think is his learning style? Circle your response:  Auditory Visual Tactile |
|  |  | 14. Write one thing you can do to meet the needs of this type of learner: |
