# Supplementary material for: Addressing Training Gaps: A Competency-Based, Telehealth Training Initiative for Community Health Workers
Source: Telemed Rep. 2023 Jun 16;4(1):126–34. doi: 10.1089/tmr.2023.0007 (PMC10282968; doi:10.1089/tmr.2023.0007)
Supplement: Supplemental data [file Suppl_AppendixTableSA2.docm]

| **APPENDIX 2.** Community Health Worker (CHW) satisfaction survey from the Texas Department of State Health Services.^9^ | |
| --- | --- |
|  | **Poor Excellent** |
| 1. Circle the number that best represents your overall rating of this program | 1 2 3 4 5 6 |
| Please evaluate the speaker(s) for the presentations: |  |
| 1. Rate the effectiveness of the instructor(s) | 1 2 3 4 5 6 |
| 1. Were the teaching strategies/methods effective? | 1 2 3 4 5 6 |
| Did the presentation meet the following objectives? |  |
| 1. Objective #1: Discuss the value of CHWs | 1 2 3 4 5 6 |
| 1. Objective #2: Recognize gaps in access to care in your community | 1 2 3 4 5 6 |
| 1. Objective #3: Define HIPAA^a^ and PHI^b^ | 1 2 3 4 5 6 |
| 1. Objective #4: Determine emergent vs non-emergent situations | 1 2 3 4 5 6 |
| 1. Were the objectives relevant to the overall purpose of the presentation? | 1 2 3 4 5 6 |
| 1. Was the venue (telemedicine) appropriate for learning? | 1 2 3 4 5 6 |
| 1. Did the presentation increase your level of knowledge in the subject? | 1 2 3 4 5 6 |
| 1. Did the presentation increase your level of interest in the subject? | 1 2 3 4 5 6 |
| 1. Was the subject matter of the course relevant to your CHW work? | 1 2 3 4 5 6 |
| 1. Describe how you will use the information presented today in your practice. | |
| 1. List topics for future education programs | |
| 1. Additional comments | |
| *^a^HIPAA. Health Insurance Portability and Accountability Act; ^b^PHI. Protected Health Information* | |
